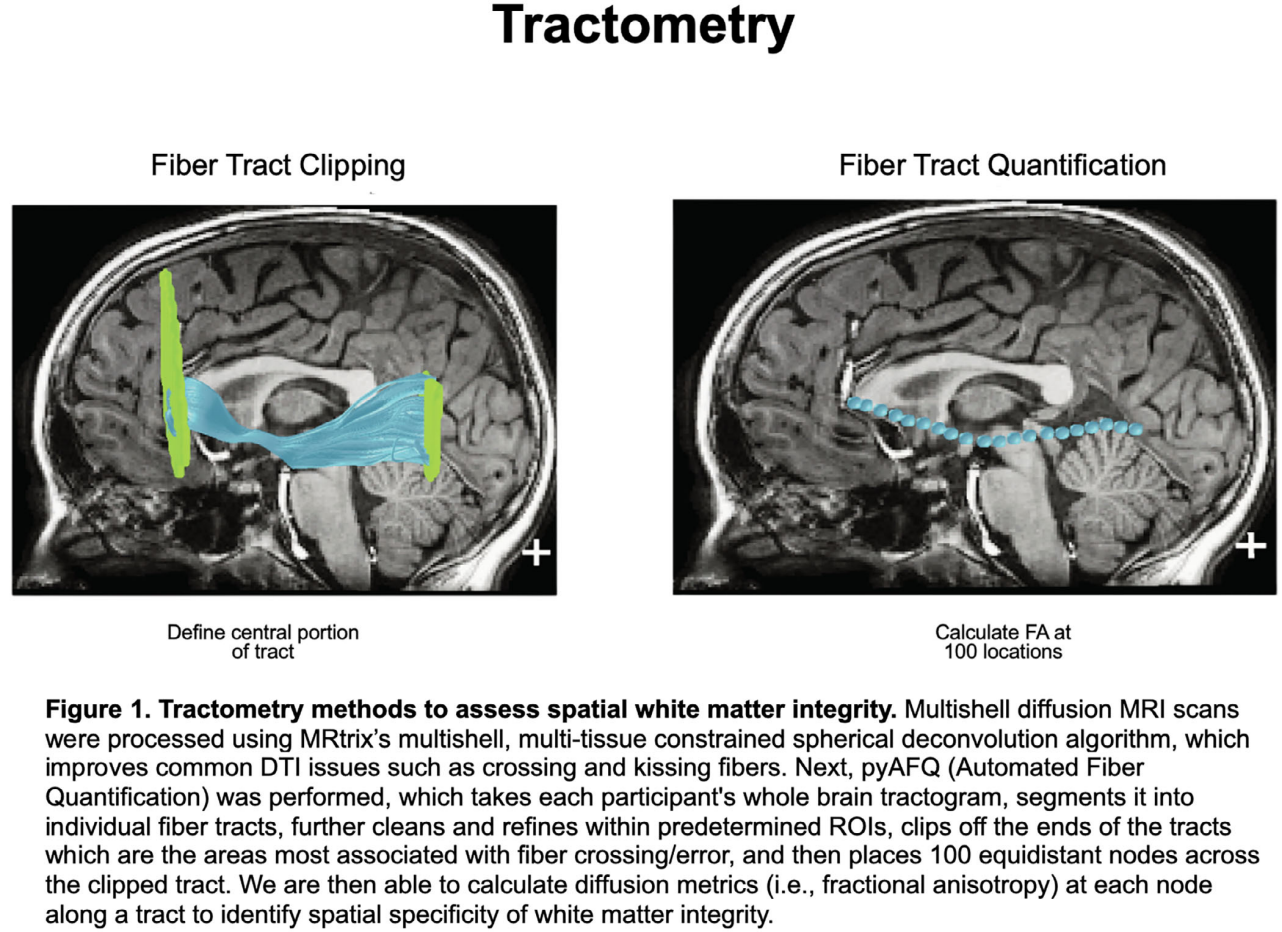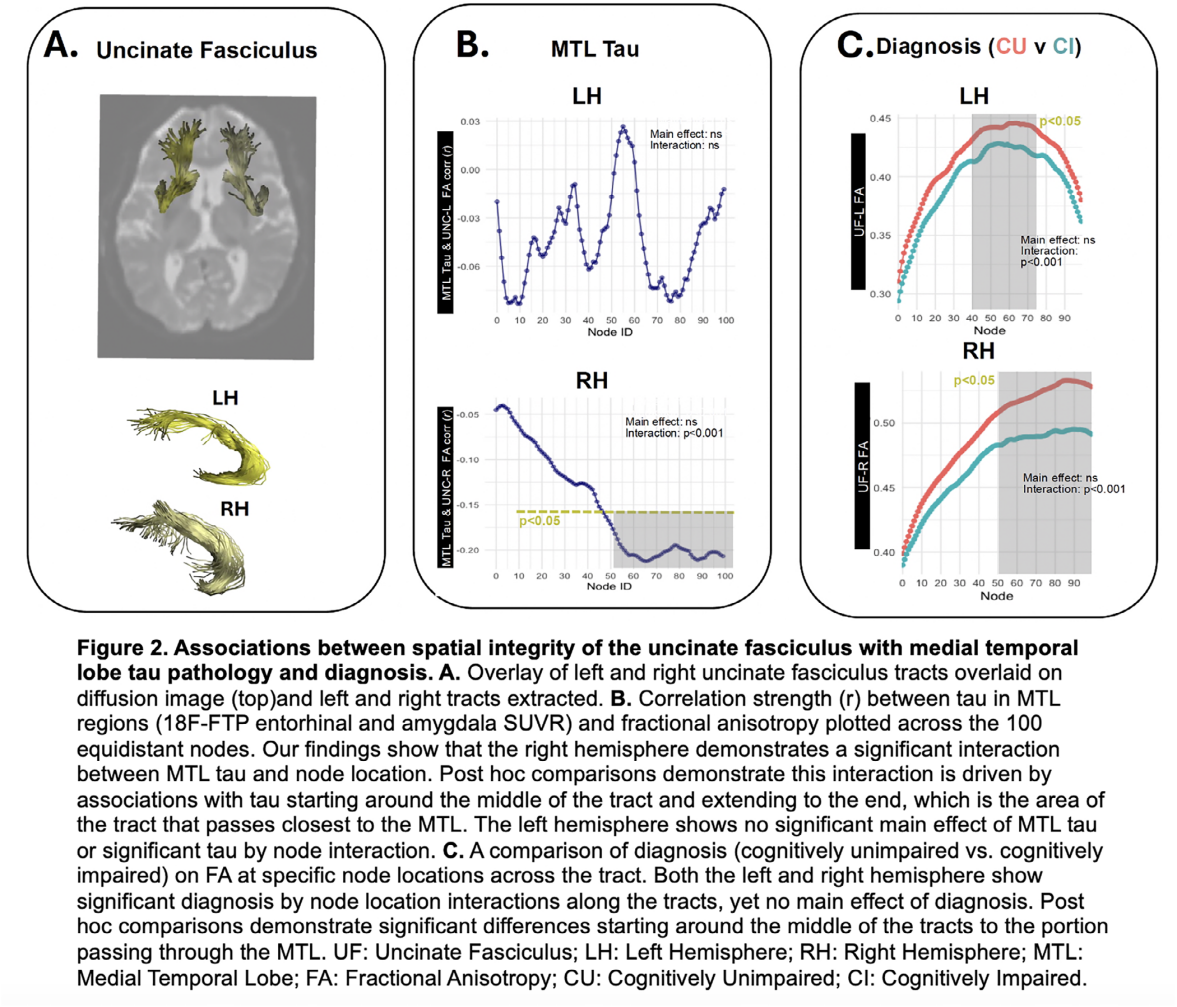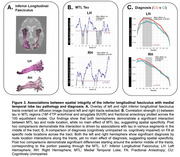# Revealing Spatial Patterns of Tau‐Related White Matter Disruption in Humans *in Vivo*

**DOI:** 10.1002/alz70856_103826

**Published:** 2025-12-25

**Authors:** Dana M Parker, Michael A. Yassa, Jenna N. Adams

**Affiliations:** ^1^ University of California, Irvine, Irvine, CA, USA

## Abstract

**Background:**

Tau pathology may contribute to the disruption of white matter (WM) integrity in Alzheimer's disease (AD). While previous work has shown global disruption to WM tracts, the specific spatial locations along the impacted tracts with disrupted integrity have yet to be identified. Tractometry is a method that utilizes diffusion MRI tractography to quantify spatial WM integrity by placing equidistant nodes along the length of WM tracts. We assessed WM integrity along the spatial extent of medial temporal lobe (MTL) WM tracts to determine tau pathology or cognitive impairment is associated with spatially specific patterns of WM integrity in older adults across the AD continuum.

**Method:**

A total of 159 older adults from ADNI3 (101 cognitively normal and 58 cognitively impaired [MCI/AD) underwent tau‐PET (18F‐Flortaucipir) and multishell diffusion MRI. Fractional anisotropy (FA), a measure of WM integrity, was calculated for each unilateral MTL tract (inferior longitudinal fasciculus [ILF], uncinate fasciculus [UF]) along 100 equidistant nodes using pyAFQ (Figure 1). Tau‐PET composite SUVRs were extracted from the entorhinal cortex and amygdala. A mixed effects model tested the main effects of tau and diagnosis on WM integrity, as well as their interactions by node to assess spatially specific effects.

**Result:**

All MTL tracts showed significant three‐way interactions between tau, diagnosis, and node location (ps<0.001), and both tau by node (ps<0.001) and diagnosis by node location interactions (ps<0.001), indicating that these factors impact WM integrity differentially along the tracts. Notably, no significant main effects of tau or diagnosis were found, highlighting the enhanced sensitivity of these spatial analyses. Post‐hoc analyses identified specific nodes within the UF (Figure 2) and ILF (Figure 3) that were significantly associated with tau levels and cognitive impairment, primarily in regions traversing the MTL.

**Conclusion:**

Our results show that assessing WM integrity along different spatial locations of WM tracts can provide more sensitive information about WM integrity in AD. Notably, these vulnerable WM nodes overlapped with areas in the MTL known for tau deposition, providing valuable insights into effects of tau pathology.